# Partial substitution of nitrogen fertilizer by Chinese milk vetch with different improvement measures achieves a win-win for rice productivity and environmental benefits

**DOI:** 10.3389/fpls.2026.1762787

**Published:** 2026-03-20

**Authors:** Shang Han, Rongyan Bu, Guopeng Zhou, Shan Tang, Hui Wang, Min Li, Wenlong Cheng, Ji Wu, Hongjian Gao

**Affiliations:** 1 College of Resources and Environment, Anhui Agricultural University, Anhui Province Key Lab of Farmland Ecological Conservation and Nutrient Utilization, Anhui Province Engineering and Technology Research Center of Intelligent Manufacture and Efficient Utilization of Green Phosphorus Fertilizer, Hefei, China; 2 Soil and Fertilizer Institute, Anhui Academy of Agricultural Sciences, Fuyang Field Observation and Research Station of Soil Quality, Ministry of Agriculture and Rural Affairs, Key Laboratory of Nutrient Cycling and Arable Land Conservation of Anhui Province, Hefei, China

**Keywords:** carbon footprint, emission reduction material, green manure, net ecosystem economic benefit, nitrogen fertilizer, water management

## Abstract

**Introduction:**

Green manuring has been proven to be a sustainable strategy for enhancing paddy soil fertility and reducing chemical fertilizer input. However, the anaerobic decomposition of green manure in flooded soils leads to substantial increase in methane (CH_4_) emissions. This study explored the coordinative effects of optimized water management and introduced emission reduction material on greenhouse gas (GHG) emissions in a Chinese milk vetch-rice rotation system.

**Methods:**

A two-year (2022–2024) field experiment was conducted with four treatments: (T1, control) winter fallow + conventional nitrogen application; (T2) green manuring + 30% nitrogen reduction; (T3) green manuring + 30% nitrogen reduction + delayed flooding; and (T4) green manuring + 30% nitrogen reduction + ethephon application. Compared to T1, T2 treatment maintained rice yields while having no significant effect on annual cumulative CH_4_ emissions from the paddy field system. The global warming potential (GWP) slightly decreased under T2 treatment due to reduced nitrous oxide (N_2_O) emissions. T3 treatment lowered CH_4_ emissions in the stage between green manuring and rice transplanting (–80.2%), leading to noticeable reductions in annual GWP (–17.6%) and GHG intensity (–23.7%). Under T4 treatment, CH_4_ emissions were suppressed after green manuring until rice harvest (–33.6%), accounting for the lowest annual GWP (–29.9%) and GHG intensity (–37.1%) alongside the highest rice yields. All improvement measures reduced the carbon footprint and enhanced the net ecosystem economic benefit, with T4 treatment demonstrating the maximum economic and environmental benefits.

**Conclusion:**

When applying Chinese milk vetch to substitute 30% of nitrogen fertilizer in the rice season, integration with delayed flooding or ethephon application leads to coordinated rice yield improvement and GHG emission reduction. This study provides a feasible agronomic strategy for green production in paddy fields and offers a technical solution for mitigating GHG emissions while safeguarding rice productivity.

## 1 Introduction

According to the Sixth Assessment Report of the Intergovernmental Panel on Climate Change (IPCC), continuous increases in atmospheric greenhouse gas (GHG) concentrations would drive global surface temperature rise until at least 2050 (Intergovernmental Panel on Climate Change, 2021). Methane (CH_4_) and nitrous oxide (N_2_O) are two powerful GHGs that make prominent contributions to climate warming. The 100-year global warming potentials (GWPs) of CH_4_ and N_2_O are respectively 25- and 298-fold higher than that of carbon dioxide (CO_2_) (Qian et al., 2023). Paddy fields represent one of the principal sources of GHG emissions, accounting for approximately 50% (CH_4_) and 10% (N_2_O) of global agricultural emissions (Wang et al., 2020). China is the world’s largest producer and consumer of rice (*Oryza sativa* L.), and rice production plays a pivotal role in national food security. In the wake of global warming, achieving high rice yields while mitigating GHG emissions from paddy fields is crucial for safeguarding food security (Yuan et al., 2019). This is also an inevitable choice for advancing agricultural green transformation, combating climate change, and fulfilling China’s commitment to ‘carbon neutrality’ by 2060.

The application of nitrogen fertilizer is an essential practice that ensures high-quality and high-yield rice production. Nitrogen application is also a major anthropogenic factor influencing GHG emissions from paddy fields (Saber et al., 2020; Liu et al., 2023). Previous studies have shown that N_2_O emissions from paddy fields are elevated with increasing level of nitrogen application in a certain range (Hu et al., 2015; Saber et al., 2020). However, the effect of nitrogen fertilizer application on CH_4_ emissions varies across different nitrogen fertilizer levels. Low levels of nitrogen fertilizer application prominently enhance CH_4_ emissions, whereas high nitrogen fertilizer levels are likely to suppress CH_4_ production (Linquist et al., 2012). Thus, a trade-off relationship may emerge between N_2_O and CH_4_ emissions from high-nitrogen soils. In the rice-growing areas of southern China, leguminous green manure crops (e.g., *Astragalus sinicus* L., also known as Chinese milk vetch, CMV) are grown in the winter fallow season and incorporated into the soil prior to rice transplanting. CMV incorporation can substitute 20–40% of nitrogen fertilizer while maintaining high and stable yields of rice (Bu et al., 2023; Gao et al., 2023). This practice has been widely recognized as an environmentally friendly and efficient sustainable fertilization pattern. However, the anaerobic decomposition of green manure in flooded soils provides abundant metabolic substrates for methanogenic archaea, thereby boosting CH_4_ emissions (Lyu et al., 2024). Hence, green manuring must be systematically optimized by integration with other agronomic management practices or technical approaches. This will enable the realization of dual goals—rice yield enhancement and GHG emission reduction in paddy fields.

In paddy ecosystems, flooding creates anaerobic soil environments, stimulating organic matter decomposition by anaerobic microorganisms (Ma et al., 2024). Methanogens use organic matter as metabolic substrate and produce CH_4_, making paddy fields a major source of atmospheric CH_4_ (Rajendran et al., 2024). Therefore, appropriate water management is considered an effective agronomic strategy for mitigating CH_4_ emissions from paddy fields (Qian et al., 2022; Li et al., 2025). Current studies have predominantly focused on water level regulation during the middle growth stage of rice (e.g., paddy drying stage) (Tariq et al., 2017; Liao et al., 2023). However, in the rice-growing areas of southern China, frequent rainfall post-transplanting potentially weakens the effect of mid-stage water management on CH_4_ emission reduction. In contrast, pre-transplanting water management, such as water restriction or intermittent irrigation before transplanting or during fallow periods, may suppress soil methanogenesis more effectively. This approach has emerged as a highly efficient and operationally feasible strategy for achieving CH_4_ emission reduction in paddy fields.

The application of GHG emission reduction materials is another potentially effective practice to mitigate GHG emissions from paddy fields amended with green manure (Chen et al., 2018). Ethylene (C_2_H_4_) is a natural phytohormone and signaling molecule that induces aerenchyma formation in rice roots (Geng et al., 2023). The aerenchyma facilitates oxygen transport to the rhizosphere soil, possibly inhibiting CH_4_ production and promoting CH_4_ oxidation (Yamauchi et al., 2014). However, well-developed aerenchyma may also provide a rapid pathway for releasing CH_4_ from the soil into the atmosphere (Aulakh, 2000). To date, C_2_H_4_ has been primarily studied as a signaling molecule in rice plants. The effect of C_2_H_4_ on GHG emissions and its applicability in paddy fields amended with green manure still lack systematic evaluation, limiting its promotion as a reliable emission reduction material for paddy ecosystems.

In this study, a two-year field experiment was conducted in a typical rice-growing area of the Yangtze River Basin. The aim of the present study was to systematically investigate the coupled effects of partial substitution of nitrogen fertilizer by CMV with optimized water management and introduced emission reduction material on rice productivity and GHG emissions from paddy fields. The results could provide empirical evidence and technical support for synergistically achieving green and high-yield rice production with enhanced environmental benefits.

## 2 Materials and methods

### 2.1 Study site

The study was carried out in a CMV-rice rotation system in Chizhou, Anhui Province, China (117°24′34′′ E, 30°38′8′′ N). The study site has a typical northern subtropical monsoon climate with annual mean precipitation of 1600 mm, sunshine of 1800 hours, and temperature of 16.5 °C. The soil type is classified as paddy soil developed from river alluvium, featuring a sandy loam texture. Prior to the experiment, the soil had a pH value of 4.8 and contained 26.78 g kg^-1^organic matter (SOM), 1.6 g kg^-1^ total nitrogen, 20.5 mg kg^-1^ available phosphorus, and 62.5 mg kg^-1^ available potassium.

### 2.2 Experimental design

The field experiment began in October 2021 and ended in May 2024. Four treatments were established: (T1, control) winter fallow with conventional nitrogen application; (T2) green manuring with 30% nitrogen reduction; (T3) green manuring with 30% nitrogen reduction and delayed flooding; and (T4) green manuring with 30% nitrogen reduction and ethephon application. In T1 treatment, 210 kg ha^–1^ of nitrogen was applied in the rice season. In T2 treatment, CMV was grown as a green manure crop to substitute 30% of nitrogen fertilizer. CMV was sown after rice harvest and then incorporated into the soil at its full-bloom stage in the following year. In T3 treatment, CMV was grown and then incorporated into the soil to partially substitute nitrogen fertilizer, with flooding delayed by 10 days. In T4 treatment, CMV incorporation was implemented alongside nitrogen fertilizer reduction and ethephon application (15 kg ha^–1^). The delayed flood duration and the application rate and timing of ethhephon were set based on previous studies (Ma et al., 2024; Sun et al., 2025). Each treatment had three replicates, and a total of 12 plots (30 m^2^ each) were arranged in a randomized block design.

The CMV variety ‘Yijiangzi’ was broadcast-sown at a rate of 30 kg ha^–1^ in late October after rice harvest. Fresh CMV biomass (22,500 kg ha^–1^) was incorporated into the soil by 20 cm deep plowing in the next mid-late May (20 days before rice transplanting). If insufficient CMV biomass was produced in a year, supplementary plant biomass was provided; if CMV biomass exceeded the target level, excessive plant biomass was mowed and removed. The actual fresh biomass incorporated was consistently close to the target across years, with the mean (± standard deviation) of 22,800 ± 750 kg ha^–1^ in 2022–2023 and 22,200 ± 810 kg ha^–1^ in 2023–2024. This ensured a relatively uniform input of organic carbon from green manure across the two experimental years. Seedlings of the local dominant rice variety ‘Quanliangyou 2118’ were transplanted at a density of 200,000 plants ha^–1^ in early June and harvest occurred in late September. After that, 30 cm of stubble was left and the remaining rice straw was completely incorporated into the soil.

No chemical fertilizers were applied in the green manure season. During the rice season, nitrogen, phosphorus, and potassium fertilizers were applied in the form of urea (46% N), superphosphate (12% P_2_O_5_), and potassium chloride (60% K_2_O), respectively. Nitrogen fertilizer was applied in three splits at basal (50%), tillering (20%), and panicle branching (30%), with a basal dose of phosphorus (75 kg P_2_O_5_ ha^–1^) and potassium (150 kg K_2_O ha^–1^) fertilizers in all plots. The cultivation practices were consistent across treatments, except that no CMV was applied in T1 treatment. Field management followed conventional cultivation practices. Pest, disease, and weed control measures were consistent with local agricultural management.

### 2.3 Gas emission sampling

GHG samples were collected using the static chamber method (Pramanik et al., 2014). The static chamber consisted of an opaque acrylic column (100 cm high, 50 cm inner diameter). A small fan and thermometer were installed at the top of the chamber to mix air and record temperature in the chamber during sampling. Following CMV incorporation, sampling was conducted at three-day intervals in the first week and then weekly thereafter. During the rice growing season, sampling was conducted at three-day intervals in the first week after each fertilization event and then weekly thereafter. If the next fertilization occurred within less than a week, additional sampling was scheduled. After rice harvest, weekly sampling was carried out. The sampling time was between 9:00–11:30 am, avoiding adverse weather conditions, such as rain or snow. The chamber was installed after the base was sealed with water, and gas samples were taken using a syringe at 0, 10, 20, and 30 min. The CH_4_ and N_2_O concentrations in gas samples were determined using a gas chromatograph (Agilent 7890B, USA).

### 2.4 Soil sampling

Before sowing CMV in 2021, a total of 15 surface soil samples (0–20 cm depth) were collected from the entire experimental field in an “S”-shaped pattern to determine the original soil properties. All collected soil samples were delivered to the laboratory, where they were air-dried and sieved. The potassium dichromate oxidation method was employed for determination of soil organic matter content. Soil total nitrogen content was quantified using the Kjeldahl method. Soil available phosphorus was extracted by 0.5 M sodium bicarbonate (NaHCO_3_) and quantified by molybdenum-antimony colorimetry. Flame photometry was performed for quantitative analysis of soil available potassium after extraction by 1 M ammonium acetate (NH_4_OAc). Soil pH was measured potentiometrically in suspensions with a water-to-soil ratio of 2.5:1 (*v*/*w*). (Bao, 2000) the day before incorporating CMV in 2023, 6 surface soil samples were collected from each experimental plot in an “S”-shaped pattern for Soil C sequestration analysis.

### 2.5 Data analysis

GHG emission fluxes were calculated using linear regression based on the rate of change in gas concentrations within the sampling chamber over time (Equation 1):

(1) F=ρ×h×(dc/dt)×[273/(273+T)]

where *F* is the gas emission flux (CH_4_: C-based, mg C m^–2^ h^–1^; N_2_O: N-based, μg N m^–2^ h^–1^); *ρ* is the gas density under standard conditions (kg m^–3^); *h* is the effective height of the sampling chamber (m); *dc*/*dt* is the rate of change in gas concentration within the chamber over time; and *T* is the mean temperature within the chamber during sampling (°C) (Liao et al., 2023).

Next, cumulative GHG emissions were estimated using (Equation 2):

(2) $$Y=\sum_{i=1}^{n}-(F_{i+1}+F_{i})/2\times (t_{i+1}-t_{i})\times 24$$

where *Y* is the cumulative gas emission (mg·m^−2^); *F* is the gas emission flux; *i* indicates the *i-*th sampling event; *t_i+_*_1_ − *t_i_* is the time interval between two sampling events (h); and *n* is the total number of sampling events.

The GWP of GHGs was obtained based on (Equation 3):

(3) $$GWP=25\times Y_{\text{CH}4}+298\times Y_{\text{N}2\text{O}}$$

where GWP represents the combined greenhouse effect of CH_4_ and N_2_O (kg ha^–1^); *Y*_CH4_ is the cumulative CH_4_ emission; and *Y*_N2O_ is the cumulative N_2_O emission (kg·ha^–1^).

The GHG intensity (GHGI) was calculated as follows (Equation 4) (Intergovernmental Panel on Climate Change, 2021):

(4) GHGI=GWP/GY

where GHGI is the potential climate impact of agricultural production per unit grain yield (kg kg^–1^) and *GY* is the actual grain yield of rice (kg·ha^–1^).

### 2.6 Calculation of carbon footprints and net ecosystem economic benefit

A carbon footprint encompasses the direct emissions of GHGs, indirect emissions from agricultural inputs, and changes in SOC storage. The area-scaled and yield-scaled carbon footprints were calculated using (Equations 5-7) (Ma et al., 2024):

(5) $$\Delta SOC=(SOC_{m}\times BD_{m}–SOC_{n}\times BD_{n})\times H\times 100$$

(6) $$Area-scaled\;CF=\Sigma (AI_{i}\times EF_{i})+25\times {\text{E}}_{\text{CH}4}+298\times {\text{E}}_{\text{N}2\text{O}}–(44/12)\times \Delta SOC$$

(7) Yield−scaled CF=Area−scaled CF/GY

where *ΔSOC* is the change in SOC storage over the two-year experimental period (kg C ha^–1^); *SOC_m_* and *SOC_n_* are the SOC content at the start and end of the experiment, respectively (g kg^–1^); *BD_m_* and *BD_n_* are the soil bulk density at the start and end of the experiment, respectively (g cm^–3^); *H* is the depth of soil sampling (0.2 m); *CF* is the carbon footprint (area-scaled: kg CO_2_-eq ha^–1^; yield-scaled: kg CO_2_-eq kg^–1^); *AI_i_* is the amount of the *i*-th agricultural input (kg ha^–1^); *EF_i_* is the emission factor for the *i*-th agricultural input (Ma et al., 2024); and *GY* is the total grain yield of rice over two years (kg·ha^–1^).

The net ecosystem economic benefit (NEEB) of rice production was calculated using (Equation 8):

(8) NEEB=Economic output–Agronomic input costs–CF cost

where economic output is the economic benefit provided by rice (CNY ha^–1^), based on the mean local market price (2600 CNY t^–1^); agronomic input costs represent the total cost of all agronomic inputs (CNY ha^–1^); and CF cost is the cost of carbon footprint (CNY ha^–1^), derived from carbon footprint (kg CO_2_-eq ha^–1^) and carbon trading price (103.7 CNY t^–1^) (Li et al., 2015).

Data processing and statistical analysis were performed using Excel 2018 (Microsoft Corp., Redmond, WA, USA) and SPSS 19.0 (IBM Corp., Armonk, NY, USA). Mean comparisons were performed using a one-way analysis of variance (ANOVA) followed by the least significant difference test (*P* < 0.05).

## 3 Results

### 3.1 Effects of different treatments on rice yield

**Figure 1:**
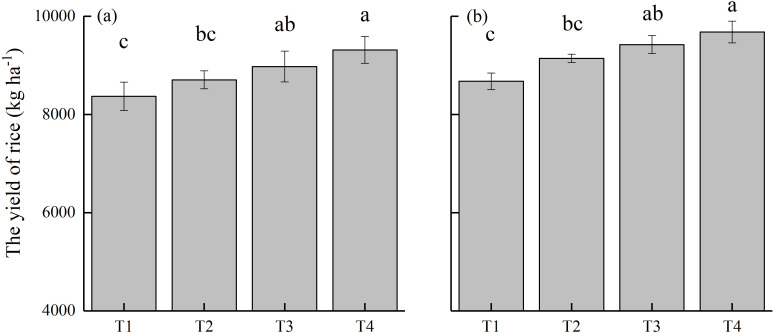
Rice yields under different treatments in **(a)** 2022 and **(b)** 2023. Error bars indicate the standard deviation of the mean (*n =* 3). Different lowercase letters on the error bars denote significant differences among treatments (*P* < 0.05). T1, Winter fallow + conventional nitrogen application; T2, Green manuring + 30% nitrogen reduction; T3, Green manuring + 30% nitrogen reduction + delayed flooding; and T4, Green manuring + 30% nitrogen reduction + ethephon application.

The annual grain yields of rice showed distinct responses to different treatments over two consecutive years (**Figure 1**). The average yield was 8369 kg ha^–1^ under conventional nitrogen application (T1, control). The substitution of CMV for 30% of nitrogen fertilizer in the rice season (T2) resulted in an average yield of 9145 kg ha^–1^, which was slightly, but not significantly, higher than that of T1 treatment. This indicates that CMV as a green manure crop partially substituted nitrogen fertilizer without reducing rice yield. The average yield reached 9425 kg ha^–1^ under CMV incorporation with delayed flooding (T3), which was significantly higher than that of T1 treatment. CMV incorporation with ethephon (T4) contributed to the highest average yield, which surpassed that of T1 (by 11.6%) and T2 (by 5.9%) treatments (*P* < 0.05). The results highlight that ethephon application further boosted rice yield on the basis of partial substitution of nitrogen fertilizer by CMV.

### 3.2 Patterns of CH_4_ and N_2_O emissions from paddy field

**Figure 2:**
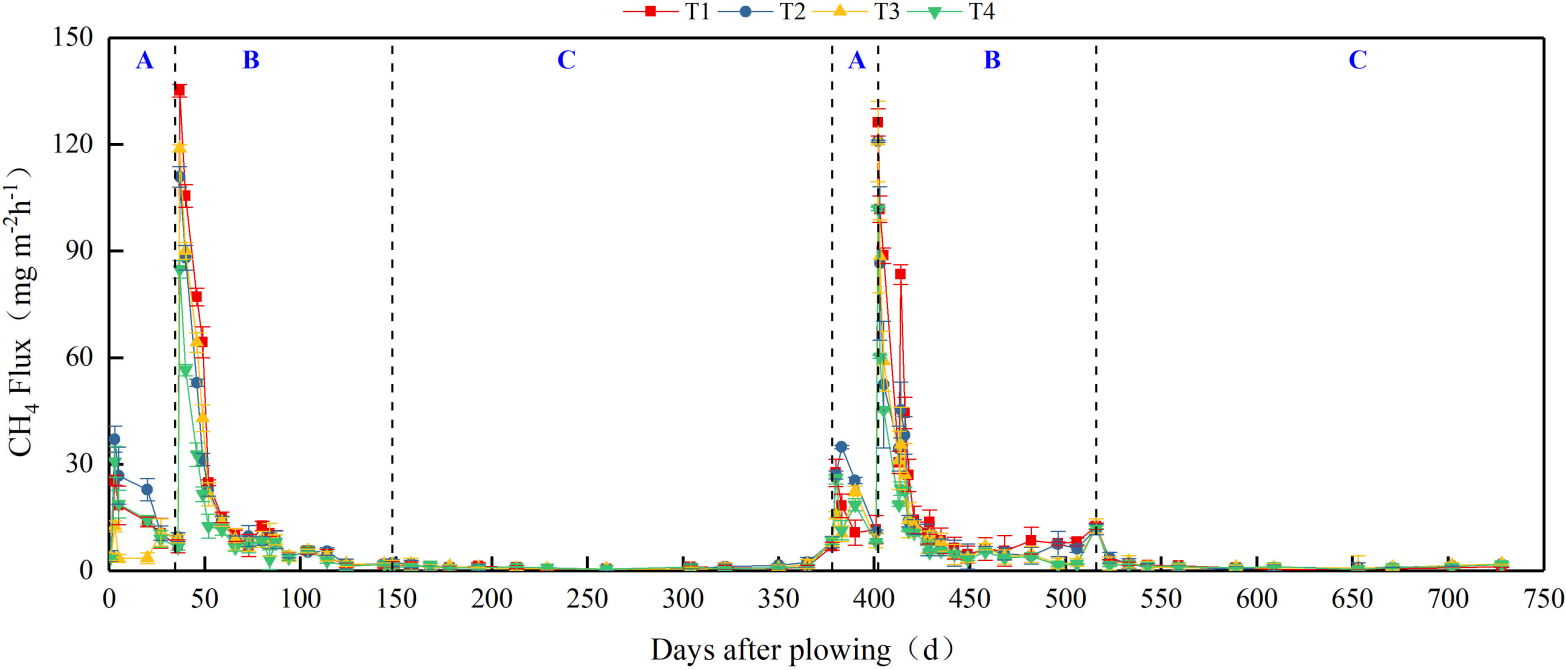
CH_4_ emission fluxes from paddy field under different treatments during 2022–2024. T1, Winter fallow + conventional nitrogen application; T2, Green manuring + 30% nitrogen reduction; T3, Green manuring + 30% nitrogen reduction + delayed flooding; and T4, Green manuring + 30% nitrogen reduction + ethephon application. **(A)** denotes the S1 stage from green manuring to rice transplanting, **(B)** denotes the S2 stage corresponding to the rice growing season, and **(C)** denotes the S3 stage from rice harvest to green manuring.

**Figure 3:**
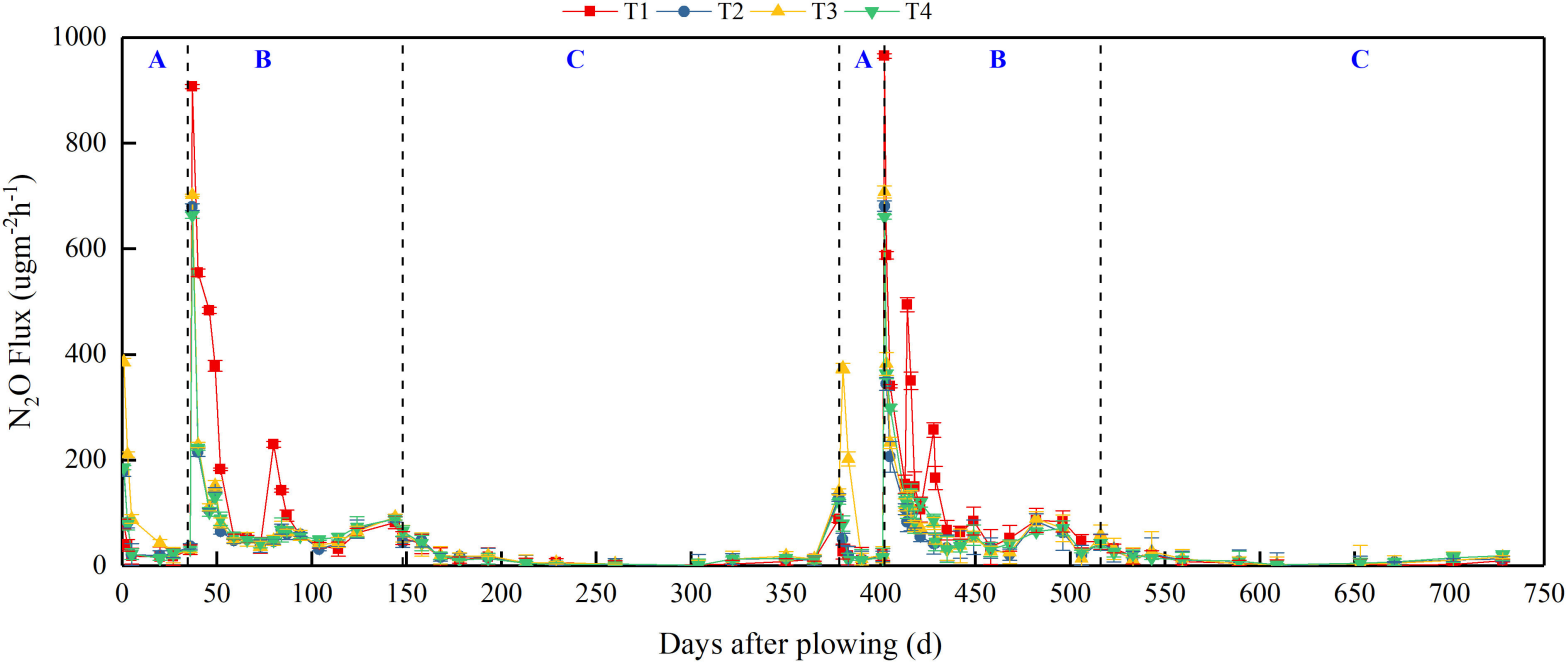
N_2_O emission fluxes from paddy field under different treatments during 2022–2024. T1, Winter fallow + conventional nitrogen application; T2, Green manuring + 30% nitrogen reduction; T3, Green manuring + 30% nitrogen reduction + delayed flooding; and T4, Green manuring + 30% nitrogen reduction + ethephon application. **(A)** denotes the S1 stage from green manuring to rice transplanting, **(B)** denotes the S2 stage corresponding to the rice growing season, and **(C)** denotes the S3 stage from rice harvest to green manuring.

**Figure 4:**
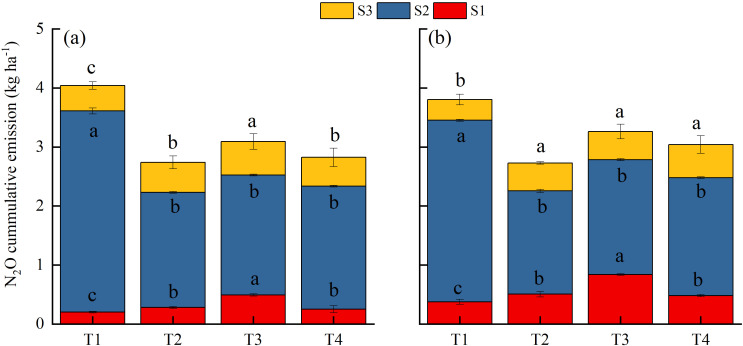
CH_4_ cumulative emissions from paddy field under different treatments in **(a)** 2022–2023 and **(b)** 2023–2024. T1, Winter fallow + conventional nitrogen application; T2, Green manuring + 30% nitrogen reduction; T3, Green manuring + 30% nitrogen reduction + delayed flooding; and T4, Green manuring + 30% nitrogen reduction + ethephon application. **(A)** is the S1 stage from green manuring to rice transplanting, **(B)** is the S2 stage corresponding to the rice growing season, and **(C)** is the S3 stage from rice harvest to green manuring. Error bars indicate the standard deviation of the mean (*n =*3). Different lowercase letters on the error bars denote significant differences among treatments (*P* < 0.05).

**Figures 2** and **3** show similar dynamics of CH_4_ and N_2_O emission fluxes from the paddy field over the two experimental years. We divided the annual cropping cycle into three stages: from CMV/rice straw incorporation to rice transplanting (S1), rice growing season (S2), and from rice harvest to CMV incorporation (S3). Under T1 treatment, the average cumulative CH_4_ emissions of individual stages accounted for 18.0% (S1), 73.4% (S2), and 8.6% (S3) of the annual cumulative emission (**Figure 4**). This emphasizes empathizes that CH_4_ emissions were primarily concentrated in the S2 stage under conventional nitrogen application. Compared to T1 treatment, substituting 30% of nitrogen fertilizer by CMV increased CH_4_ emissions in both S1 (by 43.2%) and S3 (by 17.7%) stages. However, in the S2 stage, there was a 18.2% reduction in CH_4_ emissions under T2 treatment. Thus, the annual cumulative CH_4_ emission did not differ significantly between T2 and T1 treatments. CMV incorporation with delayed flooding significantly reduced CH_4_ emissions in the S1 stage by 80.2%, with minor effects in the S2 and S3 stages. This led to a 16.3% reduction in the annual cumulative CH_4_ emission under T3 treatment compared to T1 treatment. The results reflect that CMV incorporation with delayed flooding reduced the annual cumulative emission of CH_4_ primarily by decreasing its emissions in the S1 stage. When ethephon was applied after CMV incorporation, the CH_4_ emissions of different stages were lowered by 40.1% (S1), 42.0% (S2), and 11.6% (S3), accounting for a 37.6% reduction in the annual cumulative emission. Unlike T3, T4 was an effective treatment that reduced CH_4_ emissions across all stages, resulting in the lowest annual cumulative emission.

**Figure 5:**
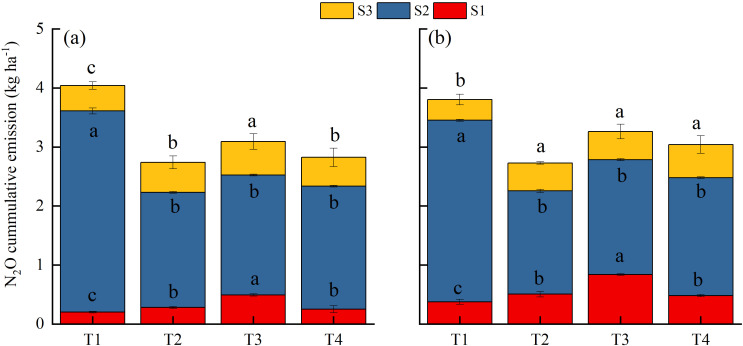
N_2_O cumulative emissions from paddy field under different treatments in **(a)** 2022–2023 and **(b)** 2023–2024. T1, Winter fallow + conventional nitrogen application; T2, Green manuring + 30% nitrogen reduction; T3, Green manuring + 30% nitrogen reduction + delayed flooding; and T4, Green manuring + 30% nitrogen reduction + ethephon application. (a) is the S1 stage from green manuring to rice transplanting, (b) is the S2 stage corresponding to the rice growing season, and (c) is the S3 stage from rice harvest to green manuring. Error bars indicate the standard deviation of the mean (*n =* 3). Different lowercase letters on the error bars denote significant differences among treatments (*P* < 0.05).

The N_2_O emission fluxes under different treatments during the experimental period are presented in **Figure 3**. Under T1 treatment, N_2_O emissions primarily occurred in the S2 stage (82.7%), with little contribution from the S1 (7.4%) and S3 (10.0%) stages (**Figure 5**). T2 treatment led to increased N_2_O emissions in both S1 (by 36.2%) and S3 (by 25.3%) stages, despite reducing emissions in the S2 stage (by 42.9%). The annual cumulative N_2_O emissions of T2 treatment were 30.3% lower than those of T1 treatment. T3 treatment elevated N_2_O emissions in both S1 and S3 stages (by 130.3% and 33.7%, respectively), with reduced emissions in the S2 stage (by 38.6%). In this case, the annual cumulative N_2_O emissions decreased by 19.0% compared to T1 treatment, despite a 14.0% rise compared to T2 treatment. Under T4 treatment, the N_2_O emissions in the S2 stage were reduced compared to those of T1 treatment, while the emissions in both S1 and S3 stages were elevated, together contributing to a 25.2% decrease in the annual cumulative emission.

### 3.3 Responses of global warming potential and greenhouse gas intensity

**Figure 6:**
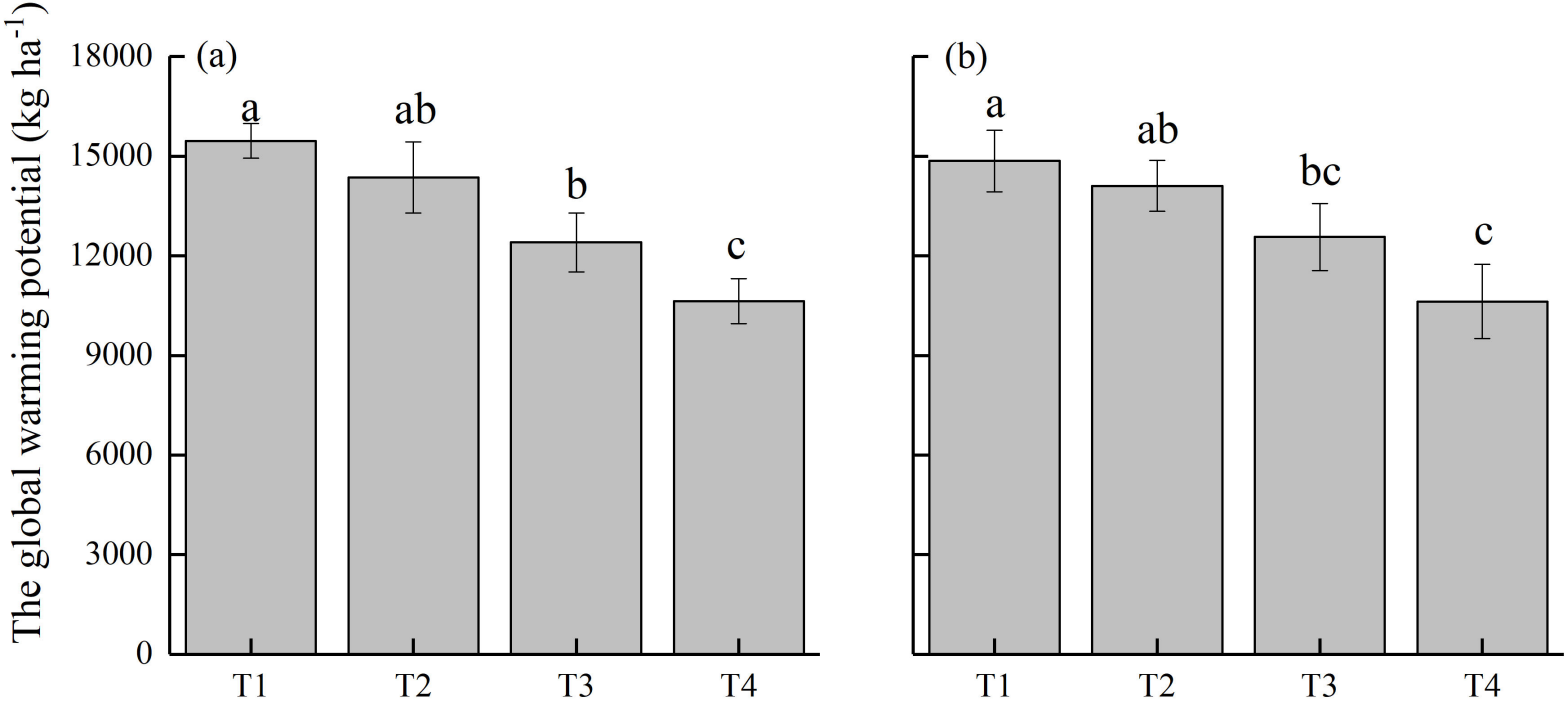
Global warming potential **(GWP)** of paddy field under different treatments during **(a)** 2022–2023 and **(b)** 2023–2024. T1, Winter fallow + conventional nitrogen application; T2, Green manuring + 30% nitrogen reduction; T3, Green manuring + 30% nitrogen reduction + delayed flooding; and T4, Green manuring + 30% nitrogen reduction + ethephon application. Error bars indicate the standard deviation of the mean (*n =* 3). Different lowercase letters on the error bars denote significant differences among treatments (*P* < 0.05).

**Figure 7:**
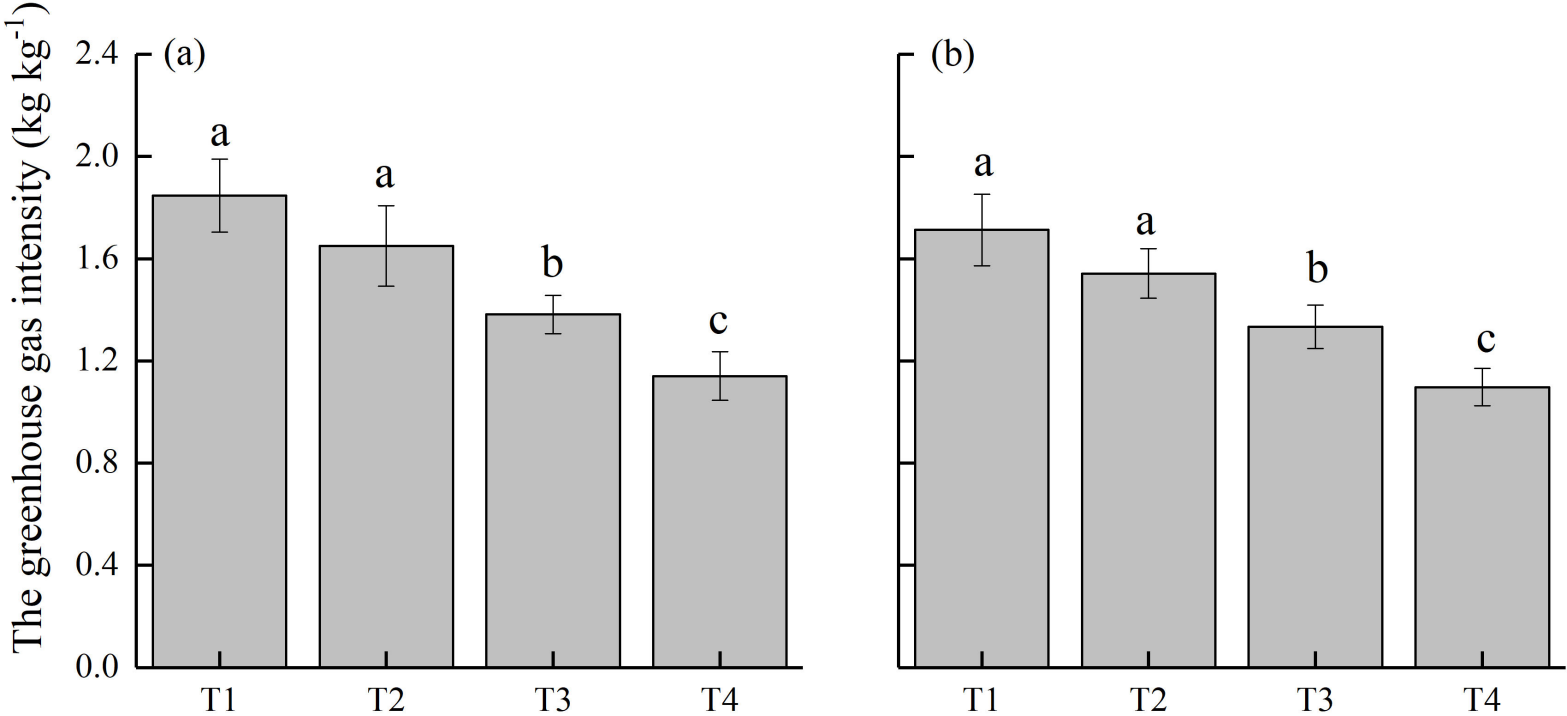
Greenhouse gas intensity (GHGI) of paddy field under different treatments during **(a)** 2022–2023 and **(b)** 2023–2024. T1, Winter fallow + conventional nitrogen application; T2, Green manuring + 30% nitrogen reduction; T3, Green manuring + 30% nitrogen reduction + delayed flooding; and T4, Green manuring + 30% nitrogen reduction + ethephon application. Error bars indicate the standard deviation of the mean (*n =* 3). Different lowercase letters on the error bars denote significant differences among treatments (*P* < 0.05).

The annual GWP values for the paddy field under different treatments ranged between 10,625–15,461 kg CO_2_-eq ha^−1^ during 2022–2024 (**Figure 6**). The highest GWP values were recorded for T1 treatment, with an average of 15,158.1 kg CO_2_-eq ha^−1^. Despite the indistinct effect of T2 treatment on CH_4_ emissions, the associated N_2_O emissions were notably lowered, resulting in a 6.1% decrease in GWP. Both T3 and T4 treatments reduced GWP (by 17.6% and 29.9%, respectively) compared to T1 treatment. In particular, the lowest GWP values were observed under T4 treatment, averaging 10628 kg CO_2_-eq ha^−1^. Further, the analysis of GHGI (**Figure 7**) revealed trends consistent with those of GWP. The peak values of GHGI were observed under T1 treatment, with 10.3%, 23.7%, and 37.1% decrease under T2, T3, and T4 treatments, respectively. The results demonstrate that the substitution of CMV for 30% of nitrogen fertilizer, alone and in combination with delayed flooding or ethephon application, diminished the GHGI of paddy field. The most prominent effect was achieved when ethephon was applied as a GHG emission reduction material.

### 3.4 Changes in carbon footprint and net ecosystem economic benefit

**Table 1** lists the carbon footprints of paddy field under different treatments. The annual area-scaled carbon footprints ranged from 0.88 to 1.39 t CO_2_-eq ha^−1^ across treatments. The carbon footprint peaked at an average of 1.36 t CO_2_-eq ha^−1^ under T1 treatment. Compared to T1, the carbon footprint was reduced under other treatments by 8.4% (T2), 21.2% (T3), and 35.0% (T4). In all cases, carbon footprint arose primarily from CH_4_ emissions (86.8–90.3%). N_2_O emissions (5.4–7.5%) and chemical fertilizers (3.4–4.6%) constituted low proportions of the annual carbon footprint. Other sources, such as seeds, pesticides, and herbicides each contributed <2.0% to the annual carbon footprint. This indicates the possibility of mitigating carbon footprint by CH_4_ emission reduction. Consistent with the pattern of area-scaled carbon footprint, the yield-scaled carbon footprint was highest under T1 treatment, followed by T2 treatment; the lowest value emerged under T4 treatment.

**Table 1:** Components of area-scaled and yield-scaled carbon footprints (CF) under different treatments during 2022–2024.

Year	Treatment	Indirect emissions (kg CO_2_-eq ha^–1^)							Direct emissions (kg CO_2_-eq ha^–1^)		ΔSOC (kg CO_2_-eq ha^–1^)	Area-scaled CF (kg CO_2_-eq ha^–1^)	Yield-scaled CF (kg CO_2_-eq kg^–1^)
		Fertilizer	Herbicide	Pesticide	Diesel oil	Rice seed	Green manure seed	Total	CH_4_ emissions	N_2_O emissions			
2022–2023	T1	666.6	7.1	3.95	36.7	55.2		769.55	14257a	1204a	2323a	13908a	1.66a
	T2	516.03	7.1	3.95	44.5	55.2	18.7	645.48	13544b	817c	2411a	12595a	1.45b
	T3	516.03	7.1	3.95	44.5	55.2	18.7	645.48	11483c	922b	2428a	10622b	1.18c
	T4	516.03	7.1	3.95	44.5	55.2	18.7	645.48	9790d	842bc	2429a	8848c	0.95d
2023–2024	T1	666.6	7.1	3.95	36.7	55.2		769.55	13721a	1134a	2373a	13252a	1.53a
	T2	516.03	7.1	3.95	44.5	55.2	37.4	664.18	13291b	813c	2481a	12288a	1.34b
	T3	516.03	7.1	3.95	44.5	55.2	37.4	664.18	11594c	972b	2456a	10774b	1.14c
	T4	516.03	7.1	3.95	44.5	55.2	37.4	664.18	9719d	906bc	2472a	8818c	0.91d

The indirect emissions were calculated based on the study of Ma et al. (2024). ΔSOC is the change in soil organic carbon storage over the two-year experimental period. T1, Winter fallow + conventional nitrogen application; T2, Green manuring + 30% nitrogen reduction; T3, Green manuring + 30% nitrogen reduction + delayed flooding; and T4, Green manuring + 30% nitrogen reduction + ethephon application.

Compared to T1, the annual agricultural input costs in the paddy field increased by 360 CNY ha^−1^ under T2 treatment (**Table 2**). However, this additional cost was offset by the environmental benefits from enhanced carbon sequestration. The NEEB values for T3 and T4 treatment exceeded those for T1 treatment (**Table 2**). Data from the two-year experiment showed that T3 and T4 treatments produced the NEEB of 18,591 and 19,557 CNY ha^−1^, respectively. These values were 10.1% and 15.8% higher than that of T1 treatment, respectively.

**Table 2:** Net ecosystem economic benefit (NEEB) of paddy field under different treatments during 2022–2024.

Year	Treatment	Agricultural input costs (CNY ha^−1^)							Economic output (CNY ha^−1^) Grain revenue	CF cost (CNY ha^−1^)	NEEB (CNY ha^−1^)
		Fertilizer	Herbicide	Pesticide	Diesel oil	Machinery	Rice seed	Green manure seed			
2022–2023	T1	835	11.2	4.8	312	1200	1500	0	21760c	1442a	16455c
	T2	722	11.2	4.8	380	1200	1500	405	22633b	1306b	17104b
	T3	722	11.2	4.8	380	1200	1500	405	23342ab	1101c	18017ab
	T4	722	11.2	4.8	380	1200	1500	405	24222a	918d	19082a
2023–2024	T1	835	11.2	4.8	312	1200	1500	0	22558c	1374a	17320c
	T2	722	11.2	4.8	380	1200	1500	405	23777b	1274b	18280b
	T3	722	11.2	4.8	380	1200	1500	405	24505ab	1117c	19165ab
	T4	722	11.2	4.8	380	1200	1500	405	25170a	914d	20032a

The rice price was 2600 CNY t^−1^. The carbon trading price was 103.7 CNY (US$17) t^–1^ CO_2_-eq (Li et al., 2015). T1, Winter fallow + conventional nitrogen application; T2, Green manuring + 30% nitrogen reduction; T3, Green manuring + 30% nitrogen reduction + delayed flooding; and T4, Green manuring + 30% nitrogen reduction + ethephon application.

## 4 Discussion

### 4.1 Co-regulation of greenhouse gas emissions by substitution of green manure for nitrogen fertilizer with water management

Based on the rice yield data from two consecutive years, substituting 30% of nitrogen fertilizer by CMV maintained stable rice production, which aligns with the result of Xie et al. (2016) and Gao et al. (2017) in southern China. This finding confirms the feasibility of green manuring to reduce nitrogen fertilizer application and safeguard food security, which represents a crucial practice for agricultural green transformation. However, the environmental impact of nitrogen fertilizer reduction, particularly concerning GHG emissions, remains highly debated.

CMV is rich in nitrogen nutrient and serves as an easily decomposable organic carbon source (Yang et al., 2019). During the S1 stage (from CMV incorporation to rice transplanting), CH_4_ emissions from the paddy field strikingly increased under CMV incorporation (T2). This result is comparable to previous findings of Kumar and Pawar (2018) and Wang et al. (2024). The decomposition of green manure could release substantial amounts of available carbon, thereby stimulating the metabolic activity of methanogens. However, during the S2 stage (rice growing season), T2 treatment led to lower CH_4_ emissions than conventional nitrogen application (T1). The possible reason is that rapid decomposition of CMV in the S1 stage already consumed a portion of readily decomposable organic carbon. This partially suppressed methanogenic activity after flooding in the rice season (Wang et al., 2020). Consequently, there was no significant difference in the annual cumulative CH_4_ emission between T2 and T1 treatments. Previously, a linear positive correlation between N_2_O emissions and nitrogen application rates in farmlands has been reported. Our results showed that the substitution of CMV for 30% of nitrogen fertilizer in the rice season notably reduced N_2_O emissions from the paddy field. However, the annual GWP and GHGI of paddy field did not differ significantly between T2 and T1 treatments, as CH_4_ dominated GHG emissions over the annual cropping cycle of the paddy field system. This result suggests that substituting 30% of nitrogen fertilizer by CMV alone is insufficient to mitigate GHG emissions from paddy fields.

Soil moisture is a crucial factor influencing GHG emissions from paddy fields. Under flooded conditions, anaerobic paddy soils provide favorable reducing habitats for methanogens (Liao et al., 2020). When incorporated into the paddy soil, CMV readily decomposed to produce substantial carbon substrates, thereby enhancing methanogenic activity and elevating CH_4_ emissions. Through delayed flooding (T3), the maintenance of aerobic soil conditions reduced CH_4_ emissions by suppressing methanogenic activity, particularly in the S1 and S2 stages. However, this treatment might simultaneously increase the potential risk of N_2_O emissions by stimulating nitrifying bacterial activity. Notably, although increased N_2_O emissions occurred under T3 treatment, the cumulative N_2_O emissions were negligible compared to CH_4_ emissions throughout the rice season. Thus, delayed flooding markedly mitigated GWP and GHGI relative to conventional water management. Consistently, Tariq et al. (2018) and Peyron et al. (2016) demonstrated marginal contribution of N_2_O emissions to overall GWP under water management, as GHG emissions from paddy fields are primarily driven by CH_4_. This result is further supported by the similar patterns of cumulative CH_4_ emissions, GWP, and GHGI in our two-year experiment. In the green manure-rice rotation system, it is difficult to achieve significant GHG emission reduction by relying solely on nitrogen fertilizer reduction. Integrating organic amendment and water management is essential for coordinately addressing the challenge of increased CH_4_ emissions.

### 4.2 Co-regulation of greenhouse gas emissions by substitution of green manure for chemical fertilizer with emission reduction material

The two-year data of rice yields showed that the substitution of nitrogen fertilizer by CMV with ethephon prominently boosted rice production, in agreement with the conclusion of Suralta and Yamauchi (2008). This effect likely stems from ethephon’s role in maintaining high root activity and facilitating nutrient uptake by the roots (Suralta and Yamauchi, 2008; Yamauchi and Nakazono, 2022). While green manuring increased CH_4_ emissions from the paddy field in the S1 stage, ethephon application considerably reduced CH_4_ emissions in both S1 and S2 stages, with the largest reduction in the S2 stage. A similar pattern has been observed by Cho et al. (2022). This emission reduction is possibly due to ethephon-mediated enhancement of oxygen transfer from the atmosphere into the soil, which promoted CH_4_ oxidation and thereby lowered CH_4_ emissions in this stage.

The monitoring results of annual CH_4_ emissions revealed that CH_4_ emissions from the paddy field were concentrated in the rice growing season (S2 stage). It has been shown that the aerenchyma in rice plants provides the primary pathway for CH_4_ emissions. Approximately 90% of CH_4_ emitted from paddy fields is released into the atmosphere through aerenchyma. Sun et al. (2025) found that C_2_H_4_ application improved the structure of aerenchyma in rice roots, thereby heightening methanotrophic activity and suppressing methanogenic activity in the rhizosphere soil. This finding provides microbiological evidence for the mechanism by which C_2_H_4_ reduces CH_4_ emissions in the rice season. When applying CMV to substitute 30% of nitrogen fertilizer in the rice season, ethephon application had little effect on N_2_O emissions. As a consequence, the annual GWP of paddy field was lowered by 33.9% after ethephon application under the experimental conditions.

### 4.3 Environmental and economic co-benefits of integrated management strategies for paddy fields

Carbon footprint is a key metric for measuring sustainable development goals (IAEG-SDGs, 2016). Assessing the carbon footprint in agricultural ecosystems paves the way for formulation of environmental policies by policymakers (Chen et al., 2020). Our results showed that substituting 30% of nitrogen fertilizer by CMV, alone and in combination with delayed flooding or ethephon application, effectively mitigated the carbon footprint of paddy field. This was largely attributed to the reduction in CH_4_ emissions. Notably, the application of CMV to substitute nitrogen fertilizer in the rice season promoted SOC sequestration. This phenomenon could be closely linked to the increase of microbial biomass carbon and the formation of recalcitrant organic carbon fractions under aerobic conditions. The dual effect on “carbon sequestration and emission reduction” unlocks possibilities for transforming paddy ecosystems from carbon sources to carbon sinks.

Comprehensive NEEB assessment provides economic justification for technology promotion. Applying CMV to substitute 30% of nitrogen fertilizer, alone and in combination with delayed flooding or ethephon application, increased the costs of green manure cultivation and CES application (ca. 810 CNY ha^–1^). Nevertheless, substantial net revenue was achieved through increased grain revenue and carbon trading revenue (103.7 CNY t^–1^ CO_2_-eq). As the NEEB advantages of T3 and T4 treatments stem from the synergistic effects of “increasing yield, reducing emissions, and saving fertilizer,” our core conclusion shows strong robustness against price fluctuations. Specifically, (1) the decline in rice price will not reverse their relative economic advantage overall; and (2) the rise in carbon price will further enhance the value of their environmental benefits. Therefore, the main conclusion holds true in various market scenarios. This finding provides a crucial reference for formulating incentive policies towards agricultural carbon emission reduction in paddy ecosystems. We suggest that T3 and T4 treatments can be applied in the rice-growing areas of the Yangtze River Basin based on site conditions. Specifically, T3 treatment is more applicable to rice-growing areas with controllable irrigation water sources because of its relatively simple technical operations and low inputs of additional materials. T4 treatment demonstrated a more sustained and pronounced effect of CH_4_ emission reduction, with a lower reliance on field water management. To ensure the stability of its emission reduction effect, we recommend applying T4 treatment in areas where strict water control is difficult to achieve due to incomplete irrigation facilities, insufficient water sources, or frequent rainfall. It should be noted that the input cost of ethephon is a factor that must be taken into consideration when choosing T4 treatment.

## 5 Conclusion

This study showed that the incorporation of Chinese milk vetch (CMV) substituted 30% of nitrogen fertilizer in the rice season without reducing rice yields, despite its indistinct effect on greenhouse gas emissions from the paddy field. Integration of CMV incorporation with delayed flooding or ethephon application not only boosted rice yields, but also reduced greenhouse gas intensity by lowering CH_4_ emissions. Delayed flooding primarily suppressed CH_4_ emissions between CMV incorporation and rice transplanting. Ethephon application decreased CH_4_ emissions from CMV incorporation to rice transplanting and throughout the rice growing season, demonstrating the optimal emission reduction effect. Both delayed flooding and ethephon application contributed to remarkable decrease in area-scaled and yield-scaled carbon footprints. These two treatments showed comparable positive effects on soil carbon sequestration and net ecosystem economic benefit. Based on the results of our two-year field experiment, we propose that CMV incorporation + nitrogen reduction + delayed flooding is suitable for rice production in areas with controllable irrigation water sources. Taking into account the CH_4_ emission effect and input cost of ethephon, CMV incorporation + nitrogen reduction + ethephon application is recommended in areas where delayed flooding is difficult to strictly implement. Future studies should refine these regional application practices by integrating long-term field experiments in different ecological zones with comprehensive cost-benefit analysis.

## Data Availability

The original contributions presented in the study are included in the article/supplementary material. Further inquiries can be directed to the corresponding authors.
